# *CreSAMT1* is mainly responsible for the biosynthesis of characteristic aroma compound dimethyl anthranilate in *Citrus reticulata* ‘Chachiensis’

**DOI:** 10.1093/hr/uhaf331

**Published:** 2025-12-04

**Authors:** Yuan Liu, Huan Wen, Zhehui Hu, Xiao Liu, Qiuhong Chen, Tinglin Wen, Yaning Liang, Yang Hu, Jiwu Zeng, Jiajing Chen, Juan Xu

**Affiliations:** National Key Laboratory for Germplasm Innovation & Utilization of Horticultural Crops, Hubei Hongshan Laboratory, College of Horticulture and Forestry Science, Huazhong Agricultural University, Wuhan 430070, China; Sensory Evaluation and Quality Analysis Centre of Horticultural Products, Huazhong Agricultural University, Wuhan 430070, China; National Key Laboratory for Germplasm Innovation & Utilization of Horticultural Crops, Hubei Hongshan Laboratory, College of Horticulture and Forestry Science, Huazhong Agricultural University, Wuhan 430070, China; Sensory Evaluation and Quality Analysis Centre of Horticultural Products, Huazhong Agricultural University, Wuhan 430070, China; National Key Laboratory for Germplasm Innovation & Utilization of Horticultural Crops, Hubei Hongshan Laboratory, College of Horticulture and Forestry Science, Huazhong Agricultural University, Wuhan 430070, China; Sensory Evaluation and Quality Analysis Centre of Horticultural Products, Huazhong Agricultural University, Wuhan 430070, China; National Key Laboratory for Germplasm Innovation & Utilization of Horticultural Crops, Hubei Hongshan Laboratory, College of Horticulture and Forestry Science, Huazhong Agricultural University, Wuhan 430070, China; Sensory Evaluation and Quality Analysis Centre of Horticultural Products, Huazhong Agricultural University, Wuhan 430070, China; National Key Laboratory for Germplasm Innovation & Utilization of Horticultural Crops, Hubei Hongshan Laboratory, College of Horticulture and Forestry Science, Huazhong Agricultural University, Wuhan 430070, China; Sensory Evaluation and Quality Analysis Centre of Horticultural Products, Huazhong Agricultural University, Wuhan 430070, China; National Key Laboratory for Germplasm Innovation & Utilization of Horticultural Crops, Hubei Hongshan Laboratory, College of Horticulture and Forestry Science, Huazhong Agricultural University, Wuhan 430070, China; Sensory Evaluation and Quality Analysis Centre of Horticultural Products, Huazhong Agricultural University, Wuhan 430070, China; National Key Laboratory for Germplasm Innovation & Utilization of Horticultural Crops, Hubei Hongshan Laboratory, College of Horticulture and Forestry Science, Huazhong Agricultural University, Wuhan 430070, China; Sensory Evaluation and Quality Analysis Centre of Horticultural Products, Huazhong Agricultural University, Wuhan 430070, China; ‘Chachiensis’ (Guangchenpi) Germplasm Resources Protection and Improved Seedling Breeding Center, Jiangmen Xinhui District Forestry Research Institute, Jiangmen 529100, China; Guangdong Fruit Institute, Guangdong Academy of Agricultural Sciences, Guangzhou 510640, China; National Key Laboratory for Germplasm Innovation & Utilization of Horticultural Crops, Hubei Hongshan Laboratory, College of Horticulture and Forestry Science, Huazhong Agricultural University, Wuhan 430070, China; Sensory Evaluation and Quality Analysis Centre of Horticultural Products, Huazhong Agricultural University, Wuhan 430070, China; National Key Laboratory for Germplasm Innovation & Utilization of Horticultural Crops, Hubei Hongshan Laboratory, College of Horticulture and Forestry Science, Huazhong Agricultural University, Wuhan 430070, China; Sensory Evaluation and Quality Analysis Centre of Horticultural Products, Huazhong Agricultural University, Wuhan 430070, China

## Abstract

*Citrus reticulata* ‘Chachiensis’ contributes its fruit peel to the raw material of ‘Guangchenpi’, is renowned for its distinctive medicinal and aromatic properties, and has been utilized for hundreds of years. However, the molecular and metabolic mechanism underlining the properties remains unknown. In this study, dimethyl anthranilate was uniquely detected in ‘Chachiensis’ fruit peel compared to other mandarin cultivars and was further validated as the characteristic metabolic biomarker based on orthogonal partial least squares discrimination analysis analysis. Two *SAMTs* genes, *CreSAMT1* and *CreSAMT2*, were screened by combined volatile profiling and transcriptome sequencing. CreSAMT1 could catalyze the methylation of N-methyl-2-aminobenzoic acid to synthesize dimethyl anthranilate, and its constant expression contributes to the specific accumulation of dimethyl anthranilate in ‘Chachiensis’, which was activated by *CreERF35* and *CreZAT11*. While *CreSAMT2* is highly expressed in citrus flowers and is responsible for catalyzing anthranilate to form methyl anthranilate, the main floral volatiles. Moreover, the involvement of transcription factors such as *ERF* were speculated in regulating its volatiles biosynthesis. The study provides a theoretical basis to elucidate the volatile metabolism, and to improve the aromatic citrus industry.

## Introduction

*Citrus reticulata* Blanco is valuable for fresh fruit and juice consumption, as well as for the medicinal properties of the peel [1–4]. Citri reticulatea pericarpium (CRP) made from the dried peel of *C. reticulata* Blanco is widely known as a traditional Chinese medicine for hundreds of years [4]. Among various raw materials of CRP, the peel of *C. reticulata* ‘Chachiensis’ (CZG) is used to generate ‘Guangchenpi’ (GCP), whereas other cultivars, such as ‘Dahongpao’ and ‘Shatangju’ (STJ), are used to generate ‘Chenpi’ (CP). GCP is considered to have superior therapeutic effects compared to CP [5–7]. Various secondary metabolites constitute the effective ingredients of GCP and perform multiple functions [8–14]. Especially, the unique volatile profile of GCP is considered to be a reliable index of its authenticity [15, 16]. The profiling of volatiles in CZG is effective to reveal the authenticity identification in GCP production [15].

Volatile compounds of citrus fruits have attracted significant attention due to their remarkable physiological functions and commercial value. Citrus fruits are rich in volatiles, including terpenoids, alcohols, aldehydes, and esters [17–21]. The volatiles are mainly stored in the oil glands of the citrus fruits flavedo in the form of essential oil [22]. Volatiles significantly contribute to citrus flavor and, consequently, influence consumer preferences [23–26]. Similar to other citrus cultivars, the terpenoids in CZG and GCP accounted for >90% of the total volatiles, with compounds such as *d*-limonene, *γ*-terpinene, and *α*-pinene being the most abundant [24, 27]. However, dimethyl anthranilate (NMM) is a specific volatile ester of CZG accounting for ~4% of the total volatiles, second only to *d*-limonene and *γ*-terpinene [28]. NMM is retained during the aging process of CZG and is subsequently preserved in GCP [15, 24, 28]. Since other mandarin germplasm such as STJ do not contain this volatile [17–19], NMM has become an important metabolite distinguishing CZG from other mandarins. Accordingly, GCP and CP made from the peels of CZG and other mandarin accessions can also be identified by NMM [15], imparting a ‘spicy’ and ‘herbal’ note and has been identified as the characteristic aroma compound of GCP, which is essential for its quality [24].

Anthranilate (AA) is an intermediate in the synthetic pathway of aromatic amino acids via the shikimic pathway [29]. The amino and carboxyl groups of AA can be methylated to form N-methyl-2-aminobenzoic acid (NMA) or methyl anthranilate (MA), respectively, while for NMM, both groups are methylated [30–33]. Methylation is a common modification process in plant secondary metabolite biosynthesis, usually mediated by methyltransferases (MTs), including those encoded by the SABATH gene family. SABATH gene family members are widespread across plant species and primarily utilize salicylic acid (SA), benzoic acid (BA), and theobromine (TH) as substrates [34, 35]. Notably, the encoded salicylic acid carboxyl methyltransferases (SAMTs) can catalyze the methylation of small acids beyond salicylic acid, such as AA and nicotinic acid [35, 36]. SAMTs in plants exhibit strong substrate heterozygosity, the PpSABATH1 and PpSABATH2 of *Physcomitrella patens* were tested for methyltransferase activity with a total of 75 compounds [37], while SAMTs in tobacco can catalyze >15 small molecular acids [35]. SAMTs have been shown to catalyze the conversion of AA into MA in maize (*Zea mays* L.), oat (*Avena* spp.), and strawberry (*Fragaria* spp.) as an odorant [32]. Furthermore, microbial engineering production of NMM has been achieved based on the characterized genes [38, 39]. However, the biosynthetic pathway of NMM in citrus has never been elucidated.

Numerous factors can affect or regulate the biosynthesis of volatiles in fruits, including transcription factors (TFs), fertilization, altitude, and exogenous phytohormone treatment [40–43]. Regulatory networks involving TFs on volatiles have been reported in citrus. For instance, the hierarchical regulation of CitMYC3 and CitAP2 promotes the accumulation of valenene in sweet orange (*Camellia sinensis*), ultimately contributing to a ‘sweet’ note [44], while CitERF71 positively regulates the accumulation of E-geraniol [45].

In this study, firstly, the volatile profile of 51 mandarin (*C. reticulata*) cultivars was analyzed via gas chromatography–mass spectrometry (GC–MS) analysis, and NMM was identified as a biomarker of CZG. Secondly, two methyltransferases, *CreSAMT1* and *CreSAMT2*, were screened through combined metabolomic and transcriptomic analysis, and were identified as functional genes involved in the NMM biosynthesis pathway, with *CreSAMT1* being considered as the major gene. Finally, the transcriptions of *CreSAMT1* and TFs of AP2 and ERF showed significant positive correlations, and dual-luc reporter assay verified that *CreERF35* and *CreZAT11* could promote the expression of *CreSAMT1*. This study provides a reference for citrus flavor breeding and theoretical foundation for quality improvement and industrial standardization of GCP.

## Results

### NMM is a unique characteristic aroma compound and biomarker of CZG

**Figure 1:**
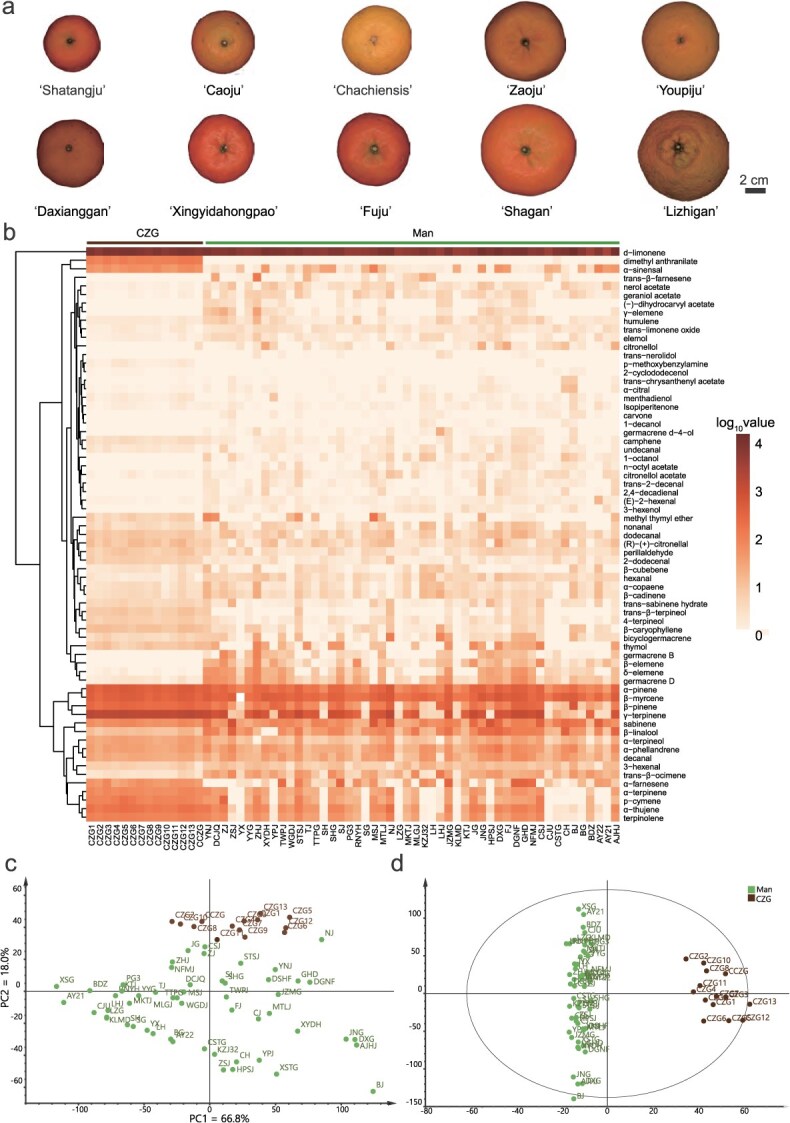
Visualization of volatile profile in different citrus cultivars. (a) Part of citrus cultivars used in this study. (b) Heatmap visualization on the differences in volatile profile of CZG and other mandarin cultivars. (c) Score scatter plot of PCA models based on the volatiles of citrus peels with the statistical parameters (PC1 = 66.8%, PC2 = 18.0%). (d) Score scatter plot of OPLS-DA models based on the volatiles with the statistical parameters (R^2^X = 0.946, R^2^Y = 0.969, Q^2^ = 0.915). ‘CZG’ represents *C. reticulata* ‘Chachiensis’, ‘Man’ represents other mandarin cultivars except CZG in this study.

GC–MS analysis revealed a total of 67 volatiles, including 49 terpenoids, 15 fatty acid derivatives, and 3 phenylpropane/benzene compounds (Fig. 1a and b, Table S1). Among all 64 mandarin accessions, *d*-limonene was the richest volatile with an average content of 8183.39 ± 2560.94 μg·g^−1^. NMM was reported as an important biomarker of CZG as well as its corresponding product GCP [5, 15, 16]. Here, NMM was found to have an average content of 118.34 ± 19.75 μg·g^−1^ in all CZG samples, while it was not detected in other mandarin cultivars. Besides, based on the published data [17], a total of 108 citrus germplasm, including orange, pomelo, lemon, and ichangensis, did not accumulate NMM in their mature peels, further demonstrating the particularity of CZG.

Principal component analysis (PCA) showed significant differences in the volatile profile between CZG and other mandarin accessions. The CZG samples clustered in the upper portion of the scatter plot, while the other cultivars were grouped in lower portion (Fig. 1c). Furthermore, the orthogonal partial least squares discrimination analysis (OPLS-DA) model distinguished the mandarin accessions more effectively (Fig. 1d). The CZG samples were clustered on the right side of the scatter plot, whereas the other mandarin accessions clustered in the center and left (Fig. 1d). The permutation test of the OPLS-DA model was conducted to exclude overfitting risks (Fig. S1). By calculating the variable importance in projection (VIP) value, volatiles contributing to sample grouping were identified. Eleven volatiles with VIP > 1.5 including NMM and *α*-sinensal were considered as unique biomarkers of CZG (Table S2), and were found to have high levels of CZG compared to other mandarin accessions. Specifically, NMM accumulated uniquely in CZG, and the average content of *α*-sinensal in CZG was 58.42 ± 12.09 μg·g^−1^, in comparison with 24.24 ± 28.09 μg·g^−1^ in other mandarin accessions.

### The accumulation of NMM differed between CZG and STJ

**Figure 2:**
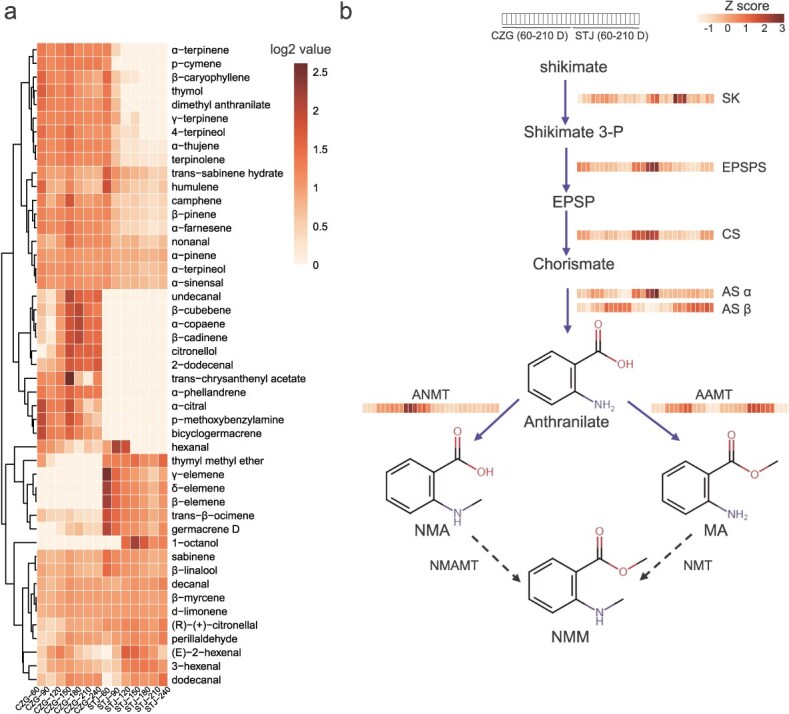
Changes in the content of NMM and the correlated biosynthetic genes. (a) Heatmap visualization on the differences in volatile profile of CZG and other mandarin cultivars. (b) The biosynthetic pathway of NMM. AAMT, anthranilic acid methyltransferase; ANMT, anthranilate N-methyltransferase; AS α/β, anthranilate synthase, AS α and AS β form noncovalent AS enzyme complexes; CS, chorismate synthase; EPSP, 5-enolpyruvylshikimate 3-phosphate; EPSPS, 5-enolpyruvylshikimate 3-phosphate synthase; MA, methyl anthranilate; NMA, N-methyl-2-aminobenzoic acid; NMAMT, N-methyl-2-aminobenzoic acid methyltransferase; NMM, Dimethyl Anthranilate; NMT, N-methyltransferase; shikimate 3-P, shikimate 3-phosphate; SK, shikimate kinase.

To further investigate the genes involved in the biosynthesis of NMM, volatiles of CZG and STJ at different developmental stages in Xinhui were analyzed. A total of 47 volatiles were identified, with 43 in CZG and 36 in STJ (Fig. 2a, Table S3). Seven volatiles including NMM that had been confirmed as important aroma active odorants in aged GCP [24] were all detected in fresh CZG peels. The contents of NMM were 895.44 ± 14.28 and 718.64 ± 82.07 μg·g^−1^ of CZG and STJ at 60 days after full bloom (DAFB), respectively. As the fruit developed, the content of NMM in STJ decreased to 23.49 ± 0.63 μg·g^−1^ at 90 DAFB and was undetectable at later stages. In contrast, NMM content in CZG remained relatively high throughout the entire fruit development process, reaching 145.91 ± 6.37 μg·g^−1^ at the physiological maturity stage (240 DAFB), when CZG is suitable for GCP production. Similarly, *γ*-terpinene was absent in STJ at maturity, while it was the second most abundant volatile after *d*-limonene in CZG, reaching 1545.88 ± 61.67 μg·g^−1^ at 240 DAFB.

### Screen candidate *CreSAMTs* via RNA-seq analysis

AA is an intermediate within shikimic acid pathway, and both its carboxyl and amino groups can be methylated to form NMA and MA, respectively, both of which are potential precursors for NMM (Fig. 2b). To identify candidate genes involved in NMM biosynthesis, a genome-wide transcript profiling analysis was conducted using RNA-sequencing on CZG peels at five developmental stages (60, 90, 120, 150, and 210 DAFB) as well as the flower, compared with those of STJ.

**Figure 3:**
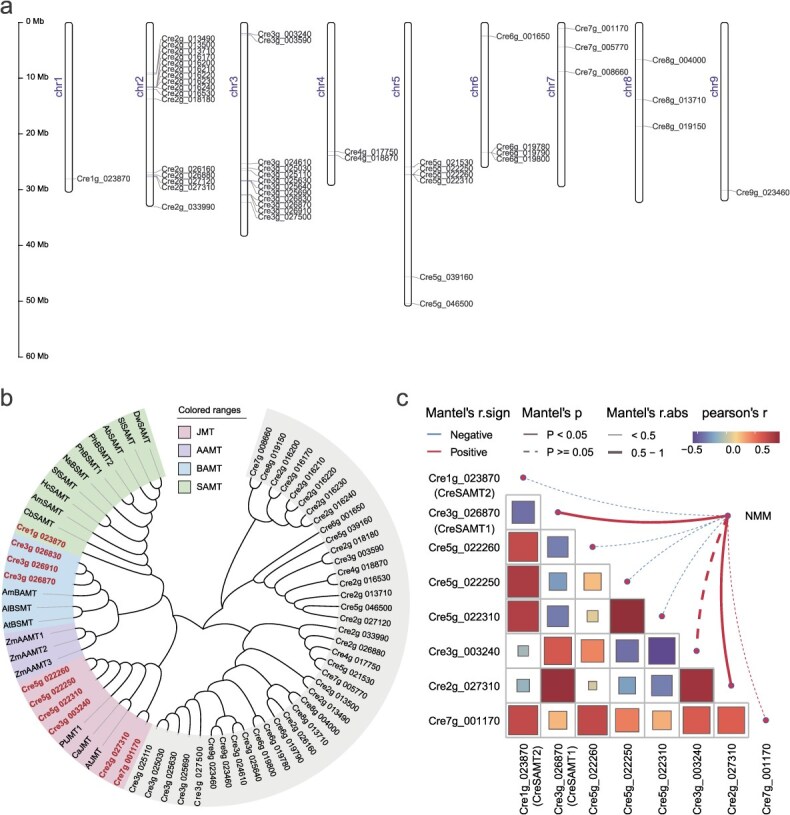
SABATH gene family related to NMM biosynthesis in CZG. (a) Schematic representation of the *C. reticulata* ‘Mangshan’ chromosomes together with the locations of candidate *CreBSMTs* genes involved in NMM biosynthesis. (b) Phylogenic analysis of candidate *CreBSMTs* and other SABATH methyltransferases involved in the methylation of small acids. The amino acid sequences were retrieved from GenBank. Ab, *Atropa belladonna* (BAB39396); At, *Arabidopsis thaliana* (AAG23343; AAY25461); Al, *Arabidopsis lyrate* (AAP57211); Am, *Antirrhinum majus* (AAN40745; AAF98284); Ca, *Capsicum annuum* (ABB02661); Cb, *Clarkia breweri* (AAF00108); Dw, *Datura wrightii* (ABO71015); Hc, *Hoya carnosa* (CAI05934); Ns, *Nicotiana suaveolens* (CAF31508); Ph, *Petunia hybrida* (AAO45012; AAO45013); Pt, *Populus trichocarpa* (AGR50489); Sf, *Stephanotis floribunda* (CAC33768); Sl, *Solanum lycopersicum* (NP_001234809); Zm, *Z. mays* (HM242244; HM242246; HM242247). (c) Correlation analysis of the *CreSAMTs* with NMM contents.

SAMTs are key enzymes for the methylation of small acids, particularly in the biosynthesis of methyl salicylate. Gene models containing PF03492 motifs, corresponding to the Methyltransf_7 domains of a typical plant *SAMTs*, were screened. A total of 49 putative *CreSAMT* genes were identified in the C. *reticulata* ‘Mangshan’ genome (Fig. 3a).

Then, a phylogenic tree was constructed using biochemically characterized SAM-dependent *SAMTs* involved in the methylation of small acids, resulting in the clustering of putative CreSAMTs into five recognized groups. The gene *Cre1g 023870.1* was clustered with other *SAMTs*, while the other three genes *Cre3g 026870.1*, *Cre3g 026910.1*, and *Cre3g 026830.1* clustered with *BAMTs* (Fig. 3b). Besides, six genes clustered with the *JMTs*, suggesting potential methyltransferase functions for other small acids. Consequently, 10 genes were considered as potential *SAMTs* involved in NMM biosynthesis, and their catalytic function remains to be verified.

RNA-seq results indicated that two of 10 candidate *CreSAMTs* were not expressed throughout the development stages of both CZG or STJ, thus they were excluded from further analysis. To further identify the key genes for NMM biosynthesis, Pearson correlation analysis was performed between the expression levels of remaining eight *CreSAMTs* and NMM accumulation profiles (Fig. 3c). Two genes, *CreSAMT1* (*r* = 0.95) and *Cre2g 027310.1* (*r* = 0.91), were found positively correlated with NMM (*P* < 0.05). Simultaneously, the system biology approach weighted gene coexpression network analysis (WGCNA) was applied to construct coexpression networks (Fig. S2), revealing that NMM is positively correlated with the ‘turquoise’ module containing *CreSAMT1*.

### Functional characterization of *CreSAMT1* and *CreSAMT2*

**Figure 4:**
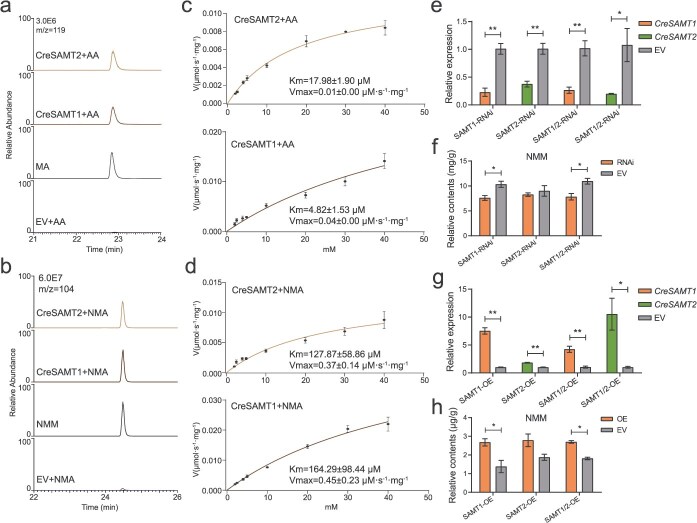
Function characterization of CreSAMTs in ‘Chachiensis’. (a and b) The catalytic function of related genes when the substrate is AA and NMA, respectively. (c and d) Steady-state kinetic analysis of CreSAMT1 and CreSAMT2 assayed with AA and NMA. The curve represents the nonlinear least-squares fit of the initial velocities versus substrate concentration to the hyperbolic Michaelis–Menten equation. *K*_m_ and *V*_max_ values are given in the plots. (e and f) The relative expression of CreSAMTs and corresponding NMM content in the CZG leaves of transient RNAi treatment. (g and h) The relative expression of CreSAMTs and corresponding NMM content in the ‘Unshiu’ leaves of transient OE treatment. Student’s *t*-test, ^*^P < 0.05, ^**^P < 0.01.

As the biosynthesis of NMM had not been previously characterized, the biochemical properties and substrate specificity of the candidate genes were analyzed. *CreSAMT1-8* were cloned and expressed with an His tag in *Escherichia coli*. Induced *E. coli* cultures were fed with SAM and six different potential acid substrates including NMA, and corresponding products were analyzed via GC–MS. Both CreSAMT1 and CreSAMT2 were shown to function as methyltransferases for all small acids (Fig. S3), while the remaining candidate genes were not. Notably, both CreSAMT1 and CreSAMT2 could methylate NMA and AA to produce NMM and MA, respectively (Fig. 4a and b).

To better understand the biochemical properties of the two enzymes, their optimal reaction conditions including temperature and pH were investigated. CreSAMT1 exhibited the highest catalytic activity at 35°C and pH = 7.0, whereas CreSAMT2 showed peak activity at 25°C and pH = 7.0 (Fig. S4). Under these optimal conditions, their kinetic properties were determined under linear reaction velocity. As shown in Fig. 4 and Table 1, CreSAMT1 demonstrated the highest affinity for AA and the highest catalytic efficiency, with the *K*_cat_/*K*_m_ value of 0.98 μM^−1^·s^−1^ for AA, while it showed the lowest substrate affinity for NMA, but its *K*_cat_/*K*_m_ is 0.19 μM^−1^·s^−1^, higher than that of BA and SA. The affinity of CreSAMT2 to the four substrates was similar to that of CreSAMT1, but its catalytic efficiency was lower, with a *K*_cat_/*K*_m_ value of 0.33 μM^−1^·s^−1^. Overall, both CreSAMT1 and CreSAMT2 exhibited the highest catalytic efficiency for AA, followed by NMA, SA, and BA.

**Table 1:** Kinetic parameters of CreSAMTs in ‘Chachiensis’

	Substrate	*K*_m_ (μM)	*V*_max_ (μM·s^−1^·mg^−1^)	*K*_cat_ (s^−1^)	*K*_cat_/*K*_m_ (μM^−1^·s^−1^)
CreSAMT1	AA	4.82 ± 1.53	0.04 ± 0.00	1.50	0.98
	BA	61.86 ± 30.72	0.03 ± 0.01	1.42	0.05
	SA	101.19 ± 33.55	0.07 ± 0.02	3.16	0.09
	NMA	164.29 ± 98.44	0.45 ± 0.23	19.05	0.19
CreSAMT2	AA	17.98 ± 1.90	0.01 ± 0.00	0.63	0.33
	BA	25.04 ± 6.89	0.01 ± 0.00	0.67	0.10
	SA	52.97 ± 11.15	0.05 ± 0.01	2.66	0.24
	NMA	127.87 ± 58.86	0.37 ± 0.14	18.32	0.31

Furthermore, both transient RNA interference (RNAi) and transient overexpression (OE) techniques were conducted to explore the actual function of *CreSAMTs* in citrus plants. The RNAi-treated CZG leaves exhibited decreased transcription levels of *CreSAMTs* (Fig. 4e). Significant reduction of NMM content was observed in the *CreSAMT1*-downregulated group (Fig. 4f). On the other hand, transient OE of *CreSAMTs* in *C. reticulata* ‘Unshiu’ leaves significantly increased the transcription levels (Fig. 4g). There was a significant increase in NMM content in the *CreSAMT1*-upregulated group (Fig. 4h). These findings demonstrated that *CreSAMT1* plays a crucial role in NMM biosynthesis in citrus.

The expression patterns of the two genes during the development of CZG and STJ were elucidated via quantitative real-time PCR (qRT–PCR) (Fig. S5). *CreSAMT1* was highly expressed during the early development stage in both cultivars. The expression level of *CreSAMT1* in STJ was almost undetectable at 120 DAFB, while its expression in CZG remained at a considerable level at 150 DAFB, which was consistent with the accumulation of NMM in different development stages in both citrus cultivars. The expression level of *CreSAMT2* in CZG was significantly higher than in STJ at 90 and 120 DAFB; however, no significant differences were observed at other stages.

Compared to NMM, the precursor AA, NMA, and MA have lower volatility, so their contents were further characterized by high performance liquid chromatography (HPLC) (Fig. S6). The content of NMA in CZG was significantly lower than that in STJ throughout the development stages, possibly because NMA is a direct precursor of NMM, and more NMA in CZG is converted to NMM compared with STJ. The content of MA in CZG was significantly lower than that of STJ at 60, 90, and 180 DAFB.

### Mining of transcription factors regulating NMM biosynthesis

**Figure 5:**
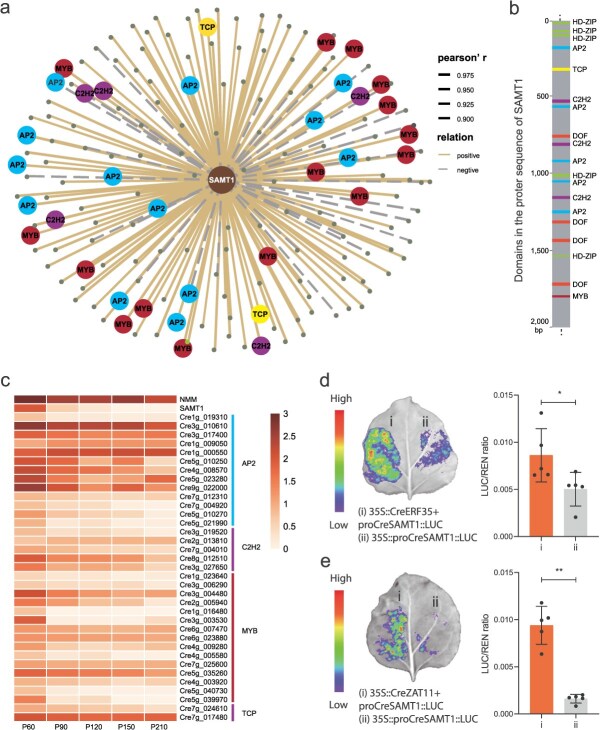
Analysis on potential TFs related to the NMM biosynthesis. (a) Coexpression network analysis based on expression patterns of *CreSAMT1*. (b) TF-binding elements identified in the promoter of *CreSAMT1*. (c) Heatmap visualization of the expression patterns among candidate TFs. (d and e) *CreERF35* and *CreZAT11* activated the promoter activities of *CreSAMT1* in a dual-luc assay, respectively.

Based on the functional characterization of candidate genes and their expression patterns during citrus development stages, *CreSAMT1* was identified as a key structural gene for NMM biosynthesis. Notably, the amino acid sequences of *CreSAMT1* in different citrus germplasm including CZG and STJ were identical. To further illustrate the regulation of NMM accumulation in CZG, a coexpression network analysis was conducted. In this analysis, the Pearson correlation coefficient between *CreSAMT1* and TFs was calculated using a correlation coefficient > 0.9 as the cut-off, and a total of 143 TFs with an average transcription levels of fragments per kilobase per million reads (FPKM) >5 were found significantly correlated with *CreSAMT1* (Fig. 5a). Most TFs showed a positive correlation with *CreSAMT1*, accounting for 83.90%. TF-binding sites, including *AP2*, *MYB*, *C2H2*, and *TCP*, etc., were notably enriched in the *CreSAMT1* promoter (Fig. 5b and c). There were 13 *AP2*, five *C2H2*, 15 *MYB*, and two *TCP* among those TFs significantly associated with *CreSAMT1*, which we focused on (Fig. 5c). Meanwhile, dual-luc reporter assay in tobacco leaves demonstrated that *CreERF35* (*Cre5g 023280*) and *CreZAT11* (*Cre7g 004010*) promoted *CreSAMT1* expression (Fig. 5d and e).

## Discussion

### NMA may be the direct precursor of NMM biosynthesis in citrus

NMM is not only a distinctive biomarker of CZG, but also serves as a key quality indicator of GCP, its corresponding downstream processing product [24, 27]. As GCP is widely applied to treat long-lasting cough and phlegm, stimulate appetite, and enhance immunity [7, 13], the identification of its bioactive substances has become a significant area of research. Multiple biochemicals like volatiles and flavonoids were found to play important parts in the health benefits of GCP [3, 7, 8]. NMM are higher in GCP aged 10 years compared to those aged three or five years [24], which implied its potential biological activity and contribution to the traditional medicinal effects of GCP. Previous studies have identified NMM as the characteristic aroma compound of GCP, highlighting the importance of understanding its biosynthesis for enhancing both CZG fruits and the GCP industry. In this study, the key structural genes involved in NMM biosynthesis were verified via multiomics analysis on CZG and STJ fruits across different developmental stages. Specifically, *SAMT1* and *SAMT2* were found to catalyze the methylation of NMA to form NMM, suggesting a potential biosynthetic pathway for NMM in citrus. In addition, MA and NMA exhibited different accumulation patterns in the two citrus cultivars. During citrus development, the content of NMA in CZG was significantly lower than in STJ, potentially due to the conversion of NMA to NMM. From a chemical standpoint, MA is a volatile compound with a low melting point, making it less likely to be stored in plants as an intermediate for long periods. In contrast, NMA is a nonvolatile compound with a higher melting point. As a result, NMA, being nonvolatile and with a higher melting point, is more stable and serves as a likely precursor for NMM biosynthesis.

### *CreSAMT1* and *CreSAMT2* are with similar function, but are synthetic genes for different volatile compounds

*CreSAMT1* and *CreSAMT2* exhibited the same broad-spectrum methyltransferase function, which can catalyze the methylation of a variety of small acids including NMA, SA, BA, and AA. However, their enzyme activities and expression patterns differed. Compared to the SAMTs genes in other plants, the enzyme activity and substrate affinity of CreSAMT1/2 in citrus are at an average level. For instance, the *K*_m_ values of AtBSMT1, AlBSMT1, and MtAAMT1 for BA are 65.00, 131.00, and 659.80, respectively [31, 34], while it was 61.86 and 25.04 for CreSAMT1/2 in the study. For SA, the SAMTs of plant species show a wide range of *K*_m_ values, among which the *K*_m_ values of GmSAMT1, AtBSMT1, and AlBSMT1 are 46.20, 16.00, and 127.00, respectively [34, 46]. While it was 101.19 and 52.97 for CreSAMT1/2 in the study, respectively. However, CreSAMT1 exhibits both a higher substrate affinity and catalytic efficiency for AA than CreSAMT2, while CreSAMT2 has a higher affinity and catalytic efficiency for BA, SA, and NMA.

Besides, *CreSAMT1* was highly expressed during the middle developmental stages of CZG, aligning with the peak accumulation of NMM. RNAi and OE experiments also support the importance of *CreSAMT1* for NMM biosynthesis in citrus. These results indicated that *CreSAMT1* was a key gene for NMM biosynthesis. In contrast, *CreSAMT2* was predominantly expressed in citrus flowers including CZG (Fig. S7), enriching the flowers with MA with fragrant note. MA is a volatile compound widely existing in the flowers of various citrus germplasm, giving citrus flowers sweet flavor [47, 48]. The unique expression pattern suggests that *CreSAMT2* is more likely the critical gene for MA biosynthesis in citrus. Secondary metabolites are active components evolved in the process of plant evolution to adapt to the external environment. They often play functions such as responding to external stimuli and enhancing plant resistance in plants. Convergent evolution and divergent evolution are common evolutionary patterns for plants to synthesize special secondary metabolites. Glycosyltransferase in the G Group in tea plants expands and undergoes functional differentiation in germplasm such as *C, sinensis* var. sinensis, playing significant roles in tea plants’ resistance to low temperature and drought stress [49], while the *GmMYBA1*/*GmMYBA2*/*GmMYBA5* genes in soybeans (*Glycine max*) also induce the accumulation of different anthocyanins [50]. *CreSAMT1/2* in citrus is tissue-specific expressed in fruits and flowers, respectively generating NMM and MA. This functional differentiation may be related to resistance and propagation, as flavorings with a ‘spicy’ note such as NMM often have the function of repelling pests and diseases, while the sweet-scented MA may play a role in attracting pollination.

### *SAMTs* contribute to flavor modification and stress resistance of citrus

Compared with other citrus cultivars, the high expression of *CreSAMT1* resulted in an accumulation of NMM in CZG with a ‘spicy’ and ‘pepper’ flavor, which can further transform into a ‘herbal’ note during the aging process in GCP [24]. Given the contribution of *CreSAMT1* to citrus flavor, this gene is considered as an important target site for citrus flavor breeding. Gene expression is influenced by various factors, such as light, temperature, and hormones [42, 51]. Jasmonate-elicited *Medicago truncatula* upregulated the expression of *MtAAMT1* through the MYB TF *MtPLATZ1*, ultimately increasing the accumulation of MA in the hairy roots of *M. truncatula* [31]. In this study, we identified several TFs such as *MYB* and *AP2* that were significantly positively correlated with *CreSAMT1* expression, while *CreERF35 and CreZAT11* were verified to promote the expression of *CreSAMT1*. These TFs are thought to be closely related to the jasmonic acid/ethylene signaling pathway in plants [52, 53]. The possible joint response of *SAMTs* genes and *ERF* TFs to stress through jasmonic acid/ethylene signaling pathway in citrus deserves further investigation. The mining of upstream TFs helped to further improve the flavor of citrus.

In addition, *SAMTs* genes play a crucial role in catalyzing the methylation of endogenous hormone SA in plants [54–57], which is involved in plant immune responses to stress [46, 58, 59]. *SAMTs* in citrus have a similar function, responding to stress factors such as Huanglongbing infection and plant hormone signals, thereby enhancing plant resistance by regulating the conversion of SA to MeSA [60]. Moreover, as a downstream product of *SAMTs*, NMM may also contribute to biological repellent for citrus plants [61, 62].

## Conclusion

In this study, the volatile profile of CZG and other mandarin cultivars were systematically compared. NMM is specifically accumulated in CZG, being a biomarker for distinguishing CZG from other cultivars. Then, the metabolome and transcriptome were combined to explore the genes related to NMM biosynthesis in CZG. Two *SAMT* genes, *CreSAMT1* and *CreSAMT2*, which can catalyze a variety of small acids, were further characterized. *CreSAMT1* is a key gene in NMM biosynthesis, catalyzing the formation of NMM from NMA. *CreSAMT2* is highly expressed in citrus flowers and is responsible for synthesizing MA, the main floral volatiles in citrus. For CZG fruits, *CreSAMT1* was constantly expressed during the whole development stage, resulting in the specific accumulation of NMM. *CreERF35* and *CreZAT11* participated in the regulation of NMM biosynthesis. This study provides a valuable reference for citrus flavor breeding and lays the foundation for improving the quality and efficiency of the downstream GCP industry.

## Materials and methods

### Samples and chemicals

All the 64 mandarin accessions used in this study were known cultivars and were collected from two citrus production areas in China, with 13 CZG cultivars at physiological maturity from Xinhui district, Guangdong Province, and 51 mandarin cultivars including CZG and STJ at physiological maturity from the Citrus Research Institute, Chinese Academy of Agricultural Sciences (Beibei, Chongqing) (Table S4). CZG fruits collected from 13 orchards under normal management in Xinhui were widely distributed in its major production areas. In addition, the CZG and STJ fruits of different developmental stages (60, 90, 120, 150, 180, 210, and 240 DAFB) were collected from Xinhui Forestry Research Institute (Xinhui, Guangdong). Leaves of CZG and *C. reticulata* ‘Unshiu’ used for gene functional characterization were collected from National Citrus Breeding Center (Wuhan, Hubei). For each cultivar, 15 fresh fruits (for three biological replicates) were collected from at least three healthy adult trees and washed with tap water. The fresh peel tissues were separated and rapidly conducted with liquid nitrogen and stored.

### Standards and reagents

GC grade methyl nonanoate (CAS No.: 1731-84-6) and methyl tert-butyl ether (MTBE, CAS No.: 1634-04-4) were purchased from Sigma Aldrich (USA). Standards for substrate feeding and protein function validation: anthranilate sodium salt form (AA, CAS No.: 552-37-4) were purchased from Aladdin (Shanghai, China); benzoic acid (BA, CAS No.: 65-85-0), salicylic acid (SA, CAS No.: 69-72-7), 3-aminobenzoic acid (3-AA, CAS No.: 99-05-8), 4-aminobenzoic acid (4-AA, CAS No.: 150-13-0), N-methyl-2-aminobenzoic acid (NMA, CAS No.: 119-68-6), methyl anthranilate (MA, CAS No.: 134-20-3), and dimethyl anthranilate (NMM, CAS No.: 85-91-6) were purchased from Sigma Aldrich (USA). S-adenosyl methionine (SAM, CAS No.: 17176-17-9) was purchased from Yuanye (Shanghai, China). All catalytic substrates and methyl donor SAM used for functional verification were dissolved in dimethyl sulfoxide, SAM was prepared as a stock solution of 500 mM, while the substrate prepared as a stock solution with a concentration of 2 M.

### Identification of volatiles

The extraction of volatiles was conducted as described [17, 24]. The qualitative and quantitative analysis of volatiles is consistent with previous study [24]. Briefly, volatiles were identified with authentic compounds and library database, while quantification was conducted based on standard curves combined with internal standard.

### Quantitative analysis of AA, NMA, and MA

The extraction of three possible precursors of NMM, AA, NMA, and MA was carried out following previously described methods [63]. For mass spectrometry data acquisition, a 1200 Series Rapid Resolution UPLC system coupled with a 1260 infinity array detector (DAD) and a QTOF 6520 mass spectrometer (Agilent Technologies, USA) was used in positive ionization mode, scanning range of 100–1700 m/z with an acquisition rate of two spectra. The UV spectra (DAD) were recorded from 200 to 600 nm. The gradient program and source conditions for electrospray ionization were adopted from our previous methods [63]. For quantification, the precursors were determined using a Vanquish HPLC system (Thermo Scientific, USA) coupled with a Hypersil GOLD C_18_ column (pore size 5.0 μm, 250 × 4.6 mm). The UV spectra were recorded from 200 to 400 nm, and the column temperature used for separation was set at 25°C. Precursors were quantified using integration areas in the calibration regression of the standards (Table S5).

### Sequence alignment and phylogenic analysis

A BLASTP search of the *C. reticulata* ‘Mangshan’ genome (http://citrus.hzau.edu.cn/index.php) was performed using the SABATH methyltransferases conserved sequence. HMM file (PF03492) for the conserved domain hidden Markov model of the SABATH gene family was downloaded from Pfam (http://pfam.xfam.org/). Different SAMTs protein sequences were aligned using the ClustalW program, and the phylogenic tree was constructed via MEGA 7 (State College, USA) base on the Neighbor-Joining statistical method.

### RNA extraction, RNA-sequencing, and qRT-PCR analysis

Total RNA of CZG and STJ peels at different developmental stages (60，90，120，150，and 240 DAFB) as well as flowers were extracted and sequenced on an Illumina HiSeq-Xten platform in Novogene. The sequencing raw data were submitted to the NCBI (PRJNA1206532, Table S6). Adapters and low-quality reads were removed followed by quality assessment using FastQC (https://www.bioinformatics.babraham.ac.uk/projects/fastqc/). The raw RNA-Seq reads of CZG and STJ were mapped on the *C. reticulata* ‘Mangshan’ genome v2.0 [64]. Uniquely mapped reads were counted with HT-seq [65].

cDNA was synthesized using HiScript II QRT SuperMix for qPCR (+gDNA wiper). qRT–PCR was performed as described [66]. The primers for qRT–PCR were specifically designed based on citrus database gene sequences (Table S7). The actin sequences were used as the reference control. The data were analyzed by using the 2^-ΔΔCt^ analysis method. qPCR data are in the form of three biological replicates with four replicates per experiment.

### Weighted gene coexpression network analysis

WGCNA analysis was performed using the corresponding package in R based on Pearson correlation coefficient [67]. The data used for WGCNA included the content of volatiles as well as the transcriptome data of CZG and STJ in five different developmental stages (60, 90, 120, 150, and 240 DAFB). The expression levels of genes were calculated using FPKM.

### Substrate feeding and *in vitro* enzyme kinetic assays in *E. coli*

The substrate feeding was carried out following previously described methods [31]. One Shot BL21 (DE3) *E. coli* cells transformed with the pET6 × HN (T7/CreSAMT1, T7/CreSAMT2) vector were used for the *in vitro* substrate feeding assay as well as the protein purification. For the substrate feeding assay, inoculate the overnight cultured expression vector strain in a 1:1000 ratio into a new 30 ml Luria–Bertani broth. The flasks were incubated at 37°C for ~4 h until the OD_600_ reached 0.6. To induce protein production, the cultures were incubated with 1 mM isopropyl *β*-*D*-1-thiogalactopyranoside at 16°C for 30 min prior to the addition of different substrate to 1 mM and incubated for 3 h. Then, the cells were extracted with 5 ml MTBE for GC–MS analysis. Relative enzyme activity with each substrate was calculated as the concentration of product, and the product that reached the highest value was set to 100%.

Besides, CreSAMT1/2 was purified from a 500-ml culture using Ni-IDA agarose and the bound proteins were eluted with 250 mM imidazole. Purified proteins were further desalted and evaluated by sodium dodecyl sulfate-polyacrylamide gel electrophoresis. The optimal reaction conditions such as pH value and temperature were tested as AA was used as the substrate. The total enzymatic reaction system included the following: protein working fluid 5 μl, 5× enzyme active buffer 10 μl (250 mM Tris–HCl, 25 mM KCl), substrate 1 μl (100–2000 mM), SAM 1 μl (500 mM), and distilled water to 50 μl. The reaction was incubated for 15 min and then extracted with 500 μl MTBE for GC–MS analysis. The temperature program of GC was set as follows: 40°C for 3 min, heated to 200°C at 5°C·min^−1^ and held for 1 min, heated to 240°C at 8°C·min^−1^ and held for 1 min, and other instrument parameters were adopted from the literature [17, 24]. Steady-state kinetic constants *V*_max_, *K*_m_, and *K*_cat_ and their errors were determined by fitting the initial velocity versus substrate concentrations including AA or NMA to the hyperbolic Michaelis–Menten equation via GraphPad Prism v8. Enzyme concentration used for the assay was calculated using Bradford’s colorimetric assay.

### Transient expression assays in citrus leaves

*Citrus reticulata* ‘Unshiu’ leaves without NMM and CZG leaves with high NMM content were utilized for transient OE and RNAi following the procedure [68] with minor modification, respectively. Five leaves were used for one replicate, and three biological replicates were designed. *Agrobacterium* strains harboring the empty vector (pK7GWIWG2D II for RNAi, and pEAQ-HT for OE) or the vector harboring the *CreSAMTs* genes were infiltrated into the equatorial region on the opposite half of each leaf. For each injection site, 0.1 ml *Agrobacterium* suspension (OD_600_ = 0.8) was used. NMM contents and transcript levels were quantified 4 days after infiltration by excising a section of leaf surrounding the injection site for both GC–MS and qRT-PCR analysis.

### Luciferase reporter assays

Luciferase (LUC) reporter assays of promoter activities were analyzed by transiently transformed *Nicotiana benthamiana* leaves as described [69, 70]. Briefly, *Agrobacterium* GV3101 (pSoup-P19) was separately transformed with reporter constructs and the empty vector. *N. benthamiana* leaves were infiltrated for 3 days with a buffered solution containing *Agrobacterium* strains. LUC activities were analyzed while luminescence values from the empty vector served as controls.

### Statistical analysis

All data were expressed as the mean value ± SD. Supervised OPLS-DA and PCA were conducted using SIMCA 14.1 (Umetrics AB, Sweden).

## Supplementary Material

Web_Material_uhaf331

## Data Availability

All data supporting the findings of this study are available within the paper and within its supplementary materials. The RNA-seq data can be found in the NCBI (PRJNA1206532).
