# Supplementary material for: CreSAMT1 is mainly responsible for the biosynthesis of characteristic aroma compound dimethyl anthranilate in Citrus reticulata ‘Chachiensis’
Source: Hortic Res. 2025 Dec 4;13(3):uhaf331. doi: 10.1093/hr/uhaf331 (PMC12968138; doi:10.1093/hr/uhaf331)
Supplement: Web_Material_uhaf331 [file web_material_uhaf331.zip › Supplementary Figures-revised.docx]

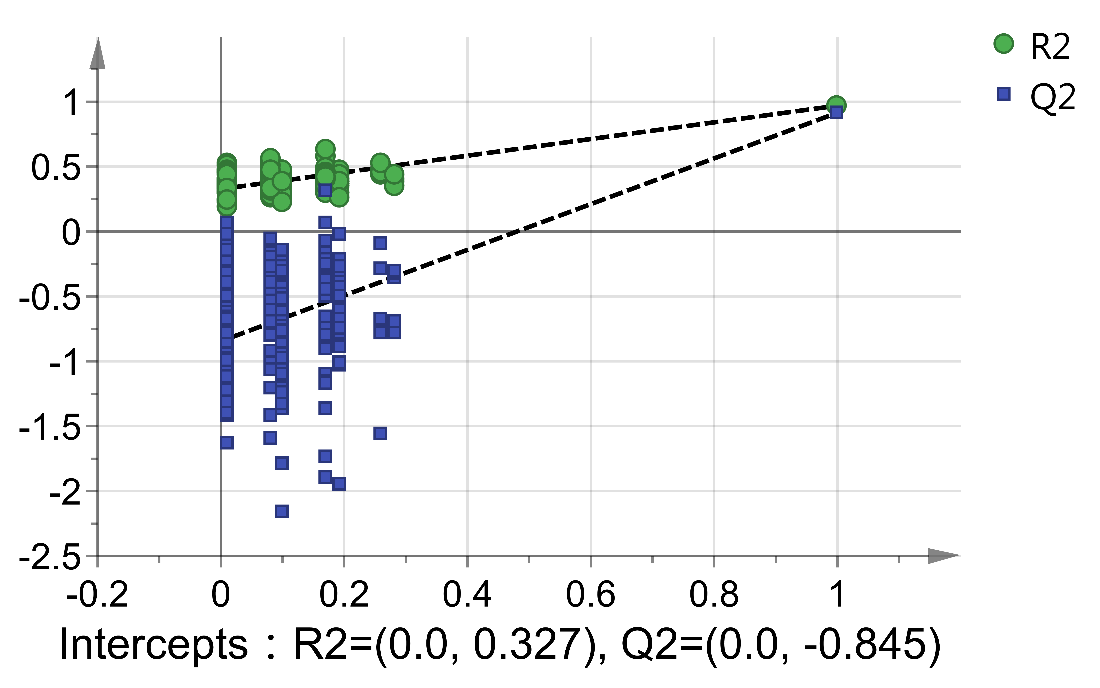


Fig S1. The permutation test of OPLS-DA model.

Intercepts of 200 permutation tests with R2 of 0.327 and Q2 of -0.845.


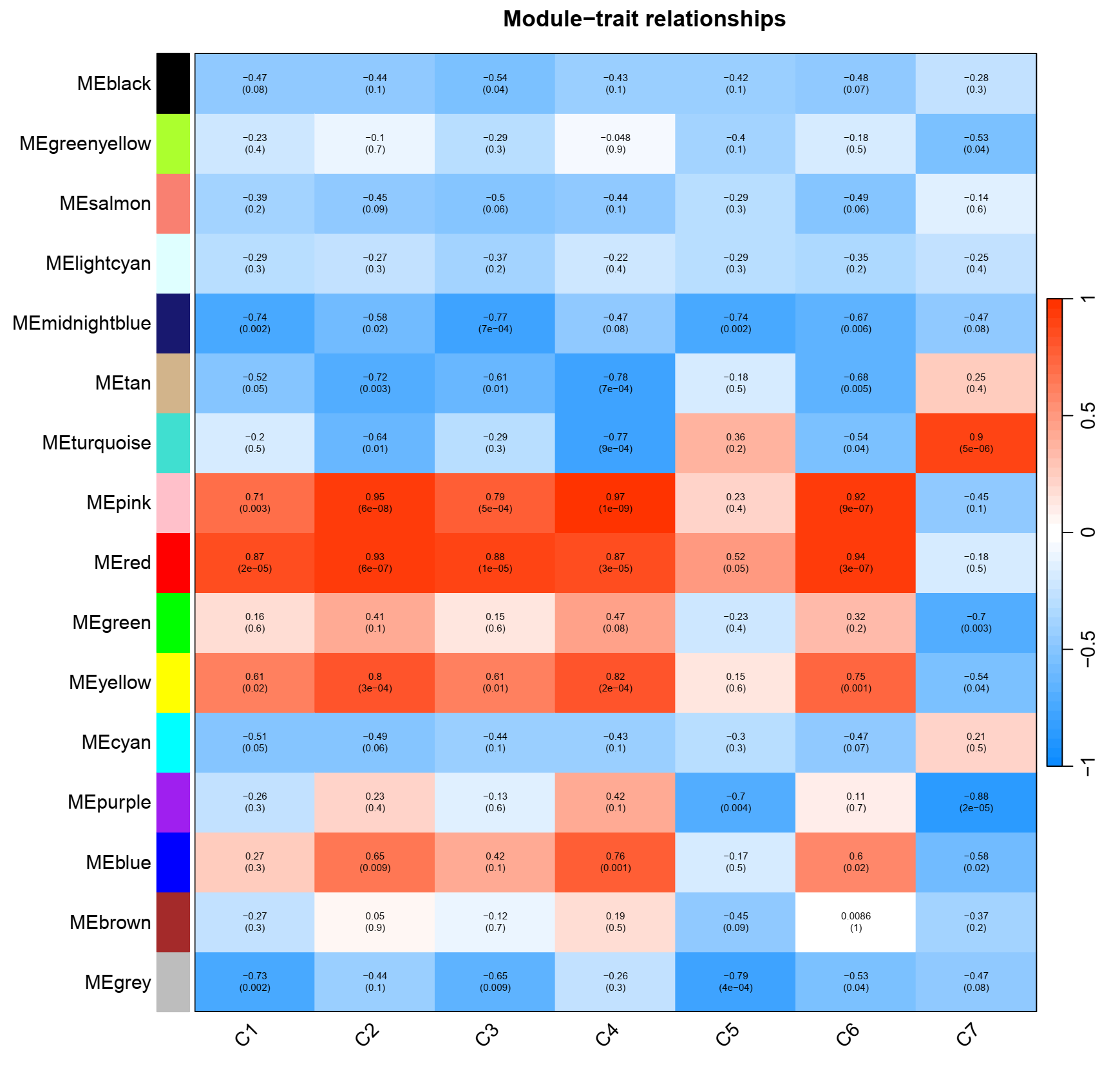


Fig S2. Gene-traits correlation heatmap of WGCNA results

C1-C7 represent *α*-pinene, *β*-myrcene, *d*-limonene, *α*-phellandrene, *γ*-terpinene, nonanal and dimethyl anthranilate respectively, which were the important odorants of CZG and GCP ^18^.


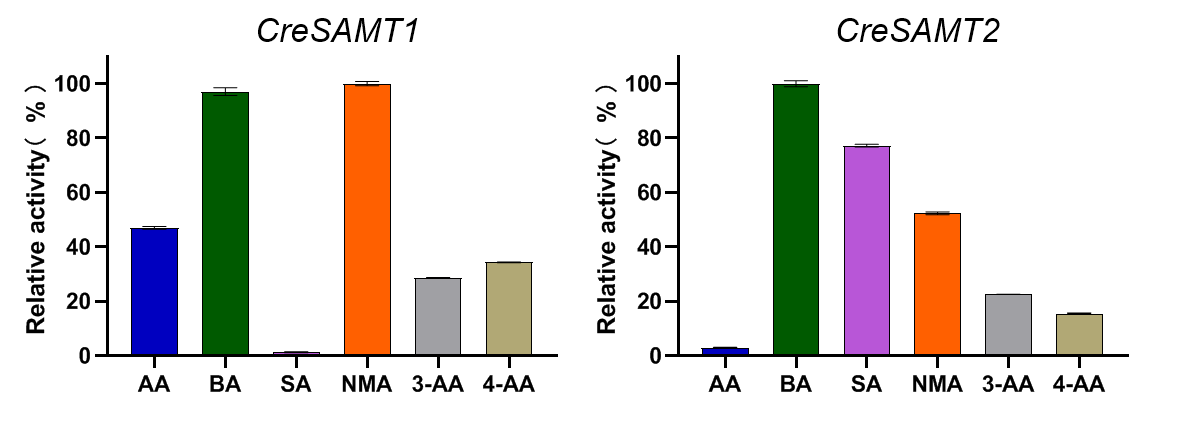


Fig S3. Function verification of *CreSAMTs* in CZG


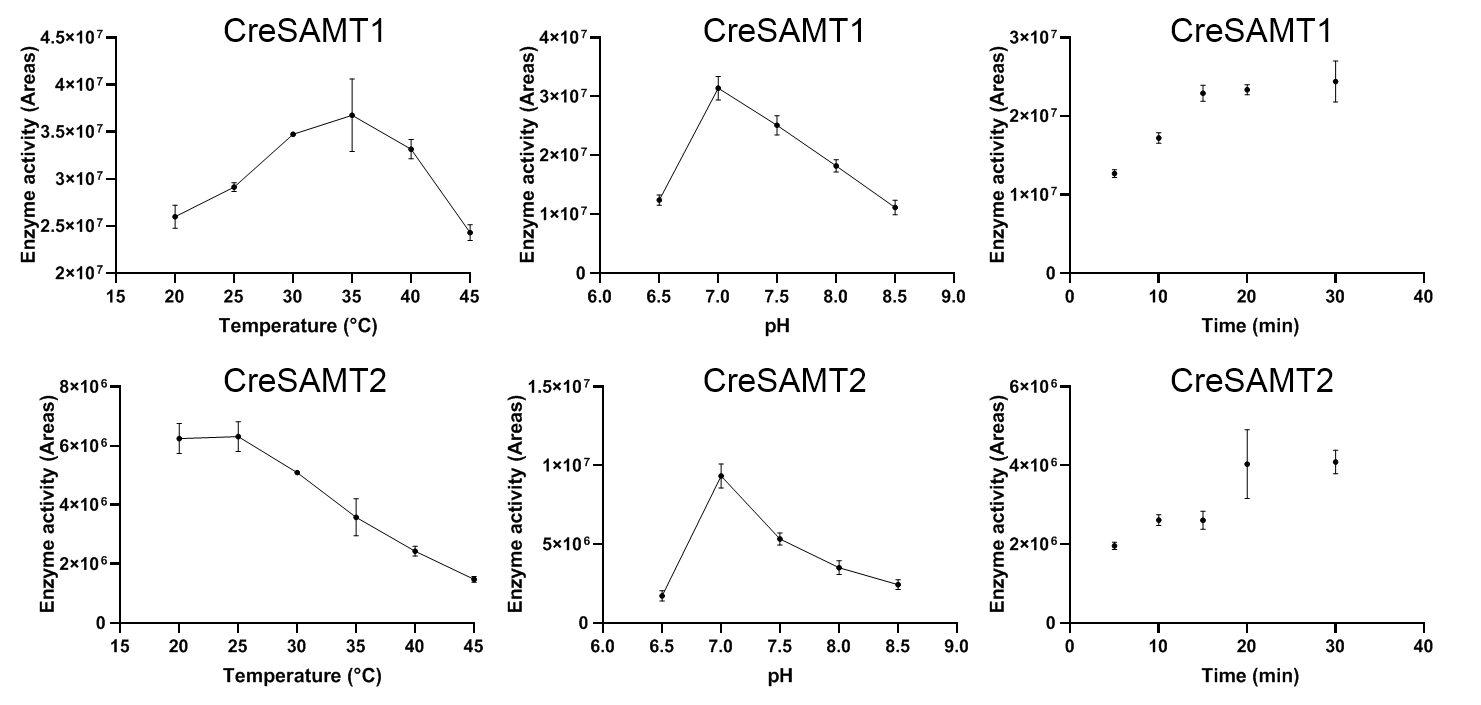


Fig S4. Catalytic activity of SAMTs under different reaction conditions


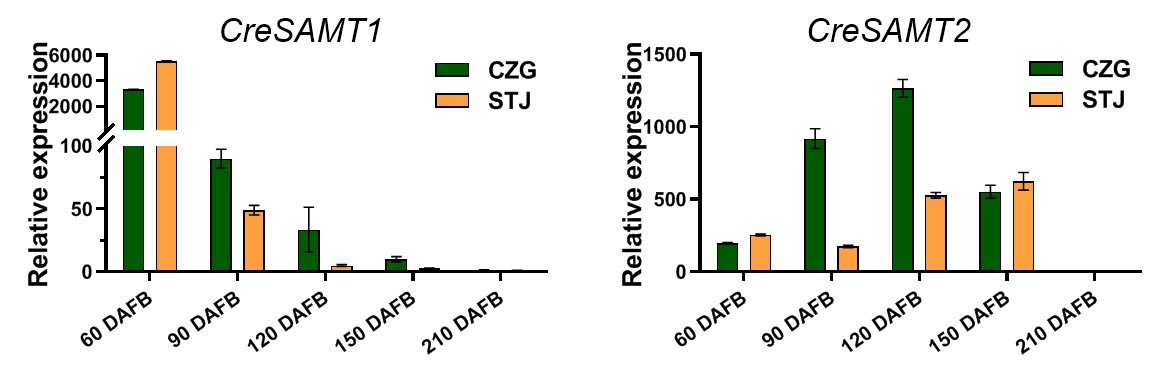


Fig S5. Relative expression levels of *CreSAMTs* in fruit development stages of CZG and STJ


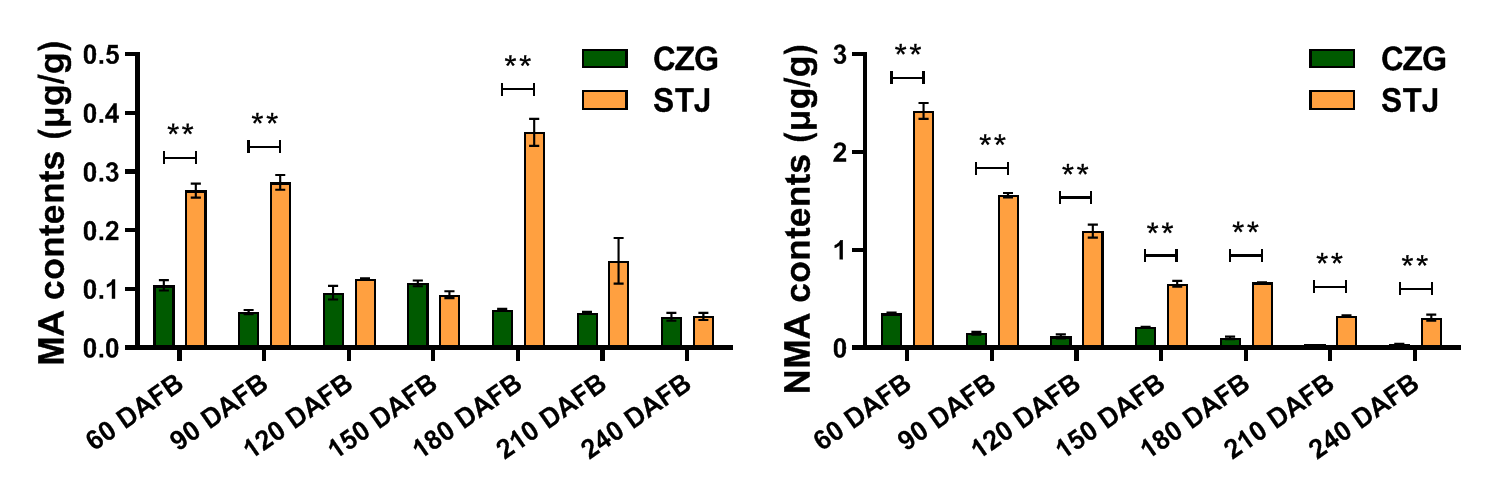


Fig S6. The changes of MA and NMA contents in fruit development stages of CZG and STJ


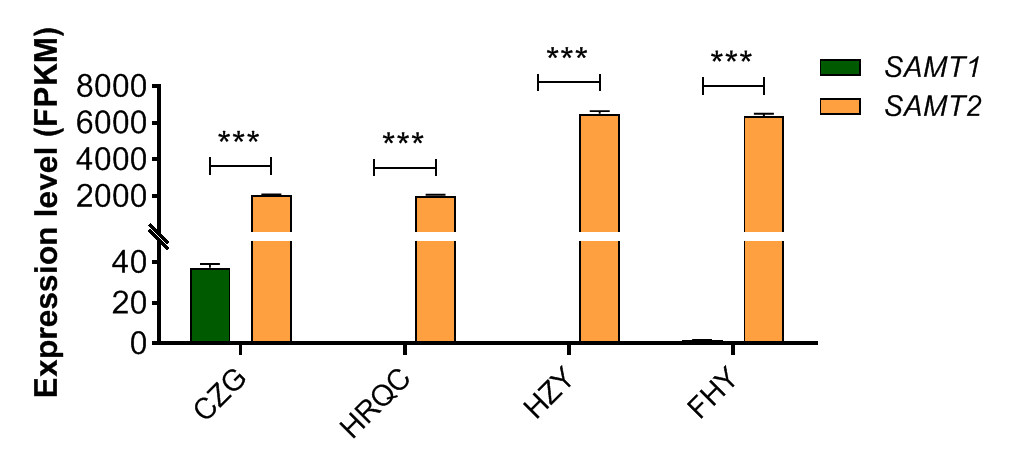


Fig S7. Relative expression levels of *CreSAMTs* in different citrus flowers
